# Economic evaluation of brivaracetam in the adjunctive treatment of patients with focal-onset seizure in Jordan

**DOI:** 10.1097/MD.0000000000045347

**Published:** 2025-10-31

**Authors:** Shiraz Halloush, Nimer S. Alkhatib, Osamah M. Alfayez, Omar S. Alkhezi, Rawan Al Froukh, Farah Radaideh, Abdallah Y. Naser

**Affiliations:** a Clinical Pharmacy and Therapeutics Department, Faculty of Pharmacy, Applied Science Private University, Amman, Jordan; b Path Economics, LLC, Amman, Jordan; c Faculty of Pharmacy, Al-Zaytoonah University of Jordan, Amman, Jordan; d Department of Pharmacy Practice, College of Pharmacy, Qassim University, Qassim, Saudi Arabia; e Department of Applied Pharmaceutical Sciences and Clinical Pharmacy, Faculty of Pharmacy, Isra University, Amman, Jordan.

**Keywords:** adjunctive therapy, antiseizure medication, brivaracetam, cost, cost-effectiveness, economic evaluation, epilepsy, focal-onset seizure, Jordan, Markov model Monte Carlo simulation

## Abstract

This study conducts a cost-effectiveness analysis of brivaracetam (BRV) compared to other 3rd-generation antiseizure medications (AEDs) for the treatment of pharmacoresistant focal-onset seizures in Jordan. A Markov model was constructed over a 2-years’ time horizon for a hypothetical cohort of focal-onset seizures patients. A cycle of 3-months was adopted in our economic evaluation (total cycles of 8 cycles). Four health states were defined: seizure free, partial responders (≥50% reduction in seizure frequency), non-responders, and discontinuation. In addition to BRV, 3 treatment comparators were included in this economic evaluation: eslicarbazepine (ESL), lacosamide (LCM), and perampanel (PER). Clinical data were retired from a previously published network meta-analysis of 65 randomized controlled trials. Cost inputs were obtained from the Jordan Food and Drug Administration and local healthcare providers. Incremental Cost-Effectiveness Ratio (ICER) was calculated using the percentage of complete response (CR) in the denominator. Probabilistic sensitivity analysis was conducted to assess the robustness of the study findings. BRV was associated with the highest gains in CR over all AEDs included in this economic evaluation. The 2-year cost of ESL is JOD 4139; LCM is JOD 3078; PER is JOD 5541; BRV is JOD 3925. The incremental gain in CR with BRV was higher by 29.0%, 30.9%, and 26.4% compared to ESL, LCM, and PER, respectively. Despite these higher gains in CR with BRV versus all other AEDs in this economic evaluation, it was associated with lower cost when compared to ESL and PER at saving ICER of -JOD 737 per 1% CR achieved and -JOD 6113 per 1% CR achieved, respectively. However, BRV was associated with the ICER of JOD 2744 per 1% of CR achieved compared with LCM. These estimates were confirmed by the probabilistic sensitivity analysis. Compared to ESL and PER, BRV was associated with cost-savings. Compared to LCM, the BRV was cost-effective at the World Health Organization recommended willingness-to-pay threshold of 3× of Jordanian gross domestic product per capita.

## 1. Introduction

Focal-onset seizures (FOS) or focal seizures, which develop from a single area in the brain, comprise 55.7% to 61.1% of all people with epilepsy.^[1]^ Oral antiseizure medication (AEDs) are the mainstay treatment for most cases of focal seizures, mostly starting with monotherapy.^[2,3]^ Seizure freedom is crucial in epilepsy treatment, however, around 64% of patients achieve it, and over one-third experience uncontrollable seizures.^[4–6]^ Uncontrolled epilepsy can cause psychological and social dysfunction, limited opportunities, and reduced quality of life, with some persistent types of seizures being a risk factor for premature death.^[4,7,8]^ Treatment-resistant patients rarely achieve seizure freedom, despite the use of newer drugs. Consequently, there are novel medications that are available to alleviate seizures in patients with uncontrolled epilepsy, thereby enabling a greater number of individuals to achieve seizure freedom.

Third-generation AEDs, including eslicarbazepine (ESL), lacosamide (LCM), perampanel (PER), brivaracetam (BRV), and zonisamide (ZNS), among others, are often used as an add-on therapy for the treatment of resistant epilepsy.^[4,9,10]^ Since 2016, evidence has developed around the comparative effectiveness of BRV versus same-class treatments, and this evidence has been demonstrated in several systematic reviews and meta-analyses.^[4,9,10]^ Such evidence was needed for formulary inclusion and reimbursement decisions across different countries in the world. For example, the pharmacoeconomic assessment conducted by Martinez et al used comparative results from meta-analyses to build on economic evidence.^[11]^ Martinez et al pharmacoeconomic evaluation found that the BRV is cost-saving and effective choice for the treatment of FOS patients compared to ESL, LCM, PER, and ZNS. However, there is no previous pharmacoeconomic evaluation in Jordan that was conducted to examine cost-effectiveness of BRV for the treatment of FOS. Such research is essential for healthcare authorities to assess the potential clinical and economic value of this new treatment for Jordanian FOS patients and, thereby, inform their decision-making process concerning formulary inclusion and reimbursement at the national level. Further, such cost-effective studies will serve as complementary tool to conventional budget impact analysis that will help decision-makers to allocate resources efficiently. Therefore, we aimed in this study to conduct a cost-effectiveness analysis of BRV compared to other third-generation AEDs for the treatment of pharmacoresistant FOS in Jordan.

## 2. Materials and methods

### 2.1. Model overview

**Figure 1.**
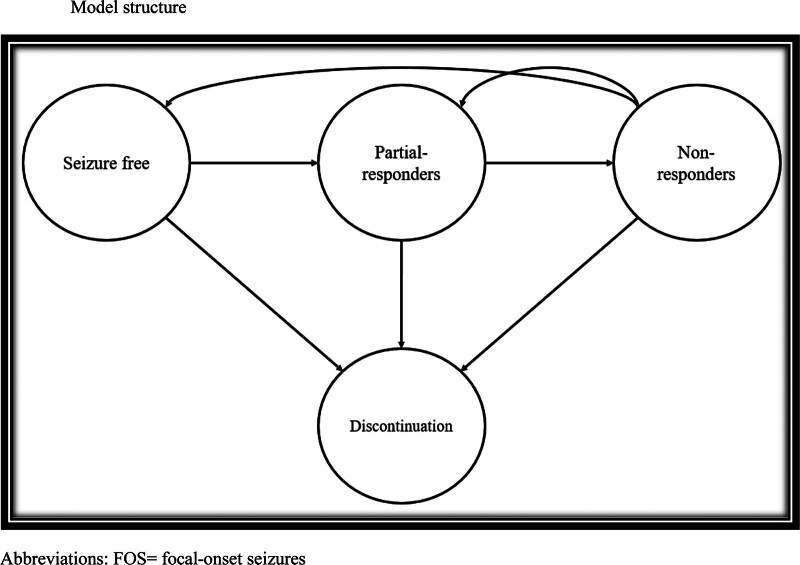
Model structure.

A Markov model was constructed over a 2-years’ time horizon for FOS patients. This time horizon is justifiable with the summary of product characteristics of BRV which limited its use to maximum 2 years.^[12]^ A cycle of 3-months was adopted in our economic evaluation (total cycles of 8 cycles) as per Martinez et al.^[11]^ Figure 1 shows the Markov model.^[11]^ Four health states were defined in this model as per Martinez et al^[11]^: seizure free, partial responders (>50% reduction in seizure frequency), non-responders, and discontinuation. Four treatment comparators were included in this economic evaluation: BRV, ESL, LCM, and PER. At the time of this evaluation, ZNS was unavailable in Jordan. Due to the limited availability of clinical data in Jordan, we used clinical trial data summarized in a previously published network meta-analysis,^[9]^ and adopted by prior pharmacoeconomic economic evaluations.^[11,13]^ Cost inputs were obtained from the Jordan Food and Drug Administration (JFDA) and local healthcare providers. This study was conducted from payer perspective.

### 2.2. Patient population and treatment dosing

Focal-onset seizures patients aged 33 to 38 years who are described in a previously published network meta-analysis that included 65 randomized controlled clinical trials formed the study population.^[9]^ This network meta-analysis summarized transitional probabilities for 11,575 FOS patients. That is, it is assumed that the results of the network meta-analysis is generalizable to the Jordanian FOS patients. The dosing of treatment comparators was based on summary of product characteristics that considers the need of FOS patients for titrating up the doses of their treatments during the first cycle. Table S1, Supplemental Digital Content, https://links.lww.com/MD/Q513 shows the median dose of treatments during the first cycle and the subsequent cycles.

### 2.3. Clinical data

In this economic evaluation, transition probability data was obtained from a previously published network meta-analysis.^[9]^ The data of comparative effectiveness between BRV and other AEDs was presented by Martinez et al in annual transition probabilities that were adjusted per trimester (3-months cycle).^[11]^ The data on risk of adverse events resulting from each treatment comparator was also derived from the same network meta-analysis.^[9]^ In the network-meta-analysis by Charokopou et al, Markov Chain Monte Carlo methods were approached to provide summary statistics and ranking of treatments.^[9,11]^ Table 1 presents the transitional probabilities of the 4 treatment comparators considered as constant across the model’s time horizon. Table 2 shows the annual probabilities for the most frequent adverse events: ataxia, dizziness, fatigue, nausea, and somnolence. Health utilities specific to each health state were not included in our study. This is because it is not available for Jordanian FOS and transferability of such patient preferences from other settings raised significant number of uncertainties. Therefore, the clinical outcome of interest in this study was the percentage (%) of patients who achieve complete response (CR), and patients have seizure-free symptoms.

**Table 1:** Model parameters.

Input	Mean estimate	SE or range	Distribution	Source
Transitional probabilities				
Responders (seizure-free)				
BRV	0.065	0.003	Beta	^[14,15]^
ESL	0.032	0.002	Beta	^[14,15]^
LCM	0.030	0.002	Beta	^[14,15]^
PER	0.035	0.002	Beta	^[14,15]^
Partial responders (50% response)				
BRV	0.355	0.018	Beta	^[14,15]^
ESL	0.330	0.017	Beta	^[14,15]^
LCM	0.308	0.016	Beta	^[14,15]^
PER	0.296	0.015	Beta	^[14,15]^
Non-responders (1-(other probability)				
BRV	0.370	0.011	Beta	^[14,15]^
ESL	0.469	0.009	Beta	^[14,15]^
LCM	0.448	0.011	Beta	^[14,15]^
PER	0.493	0.009	Beta	^[14,15]^
Discontinuation (any reason)				
BRV	0.210	0.011	Beta	^[14,15]^
ESL	0.169	0.009	Beta	^[14,15]^
LCM	0.214	0.011	Beta	^[14,15]^
PER	0.176	0.009	Beta	^[14,15]^
Cost				
BRV (per mg)	JOD 0.042	0.016	Gamma	^[16]^
ESL (per mg)	JOD 0.004	0.004	Gamma	^[16]^
LCM (per mg)	JOD 0.013	0.003	Gamma	^[16]^
PER (per mg)	JOD 0.804	0.267	Gamma	^[16]^
Adverse event management				
Acetazolamide (per mg)	0.001	0.001	Gamma	^[16]^
Cinnarizine (per mg)	0.001	0.001	Gamma	^[16]^
Health care services				
Inpatient care (1 night per event)	JOD 100	±25%	Gamma	Local hospital
Emergency visit	JOD 30	±25%	Gamma	Local hospital
Outpatient visit to neurologist	JOD 14	±25%	Gamma	Local hospital
GP visit	JOD 8	±25%	Gamma	Local hospital
EEG	JOD 100	±25%	Gamma	Local laboratory
Discounting rate				
Clinical outcomes and cost outcomes	3.50%	0%, 5%	Beta	^[17]^

BRV = brivaracetam, ESL = eslicabazepine, GP = general practitioner, LCM = lacosamide, PER = perampanel.

**Table 2:** Annual probabilities of adverse events.

Safety	BRV	ESL	LCM	PER
Ataxia	0.030	0.060	0.080	0.040
Dizziness	0.130	0.230	0.240	0.230
Fatigue	0.110	0.060	0.060	0.070
Nausea	0.050	0.120	0.110	0.010
Somnolence	0.140	0.120	0.100	0.14

BRV = brivaracetam, ESL = eslicabazepine, LCM = lacosamide, PER = perampanel.

### 2.4. Economic data

Table 1 shows the treatments cost which was based on the JFDA public price.^[14]^ The calculation was standardized to per mg because all AEDs, except ESL, are available in different doses and forms. Therefore, we calculated the cost per mg to estimate the cost of 90 days of each trimester. The costs of inpatient care, emergency visit, outpatient, general practitioner visit, and electroencephalogram (EEG) were elicited from local hospitals and laboratories. The model was intended to accommodate a ±25% variation in the cost inputs, as all costs utilized in the model are subject to some degree of uncertainty.

In this economic evaluation, patients who suffered from ataxia are assumed to receive acetazolamide; patients who suffered from dizziness and nausea are assumed to receive cinnarizine; patients who suffered from fatigue and somnolence are assumed to have no intervention. These treatments were recommended by the JFDA (17). While some recommend reducing the daily dose of treatments, the analysis assumed the wastage scenario to consider for possible wastages drug cost in case of dose reduction. The cost of adverse events management was calculated per mg are shown in Table 1 and were obtained from the JFDA.^[14]^ In each health state identified in this economic evaluation, healthcare monitoring and follow-ups were implemented for FOS patients. In each trimester, FOS patients in the seizure-free and partial response states were assumed to have at least 1 outpatient visit, and an EEG test. It is presumed that non-responding patients have been admitted to an emergency department and hospitalized for a minimum of 1 night, which should be followed by outpatient visits and an EEG test. Patients who discontinued the treatment were assumed to have only outpatient visit. Any patient who has an adverse event was assumed to visit the nearest general practitioner. All these assumptions were validated by expert clinicians served in the JFDA pricing committee.

### 2.5. Statistical analysis

The main outcome of this study was to estimate the Incremental Cost-Effectiveness Ratio (ICER) for BRV compared to other third-generation AEDs for the treatment of pharmacoresistant FOS. The numerator contained the incremental cost of BRV in comparison to any of the comparators, while the denominator contained the incremental gains in the percentage of CR from BRV and any treatment comparators. The ICER was estimated in pairwise comparison that uses BRV as a mutual comparator to all other AEDs included in this study. All ICER estimates were estimated as deterministic ICERs that are based on mean values of the model inputs; and as probabilistic ICERs that resulted from probabilistic sensitivity analysis (PSA). In the PSA, 2000 simulations were performed using a Monte Carlo second-order simulation, simultaneously changing the parameters within pre-specified distributions. The confidence interval around probabilities was distributed using beta distributions; costs were assumed to follow gamma distribution by considering variations of ± 25%. In this economic evaluation, a discount rate of 3.5% (range in PSA = 0% to 5%) was applied on both clinical and economic outcomes resulting in the second year of the model. This was based on the Central Bank of Jordan estimation.^[15]^ The ICER plane was generated and cost-effectiveness acceptability curve was plotted to evaluate the certainty of BRV being cost effective at the World Health Organization recommended 3× gross domestic product per capita as a willingness-to-pay (WTP) threshold.^[18]^ The WTP was estimated in Jordan at JOD 9000 per treatment effect. All analyses were performed in Microsoft Excel 365 (Redmond) with MSO supported coding of Visual Basic for applications.

## 3. Results

### 3.1. Base case analysis

#### 3.1.1. Deterministic ICER

As shown in Table 3, the deterministic analysis showed that the BRV was associated with the highest gains in CR over all AEDs included in this economic evaluation. The incremental gain in CR with BRV was higher by 29.0%, 30.9%, and 26.4% compared to ESL, LCM, and PER, respectively. Despite these higher gains in CR with BRV versus all other AEDs in this economic evaluation, BRV was associated with lower cost when compared to ESL and PER at saving ICER of -JOD 737 per 1% CR achieved and -JOD 6113 per 1% CR achieved, respectively. However, BRV was associated with the incremental ICER of JOD 2744 per 1% of CR achieved compared with LCM.

**Table 3:** Deterministic results and probabilistic sensitivity analysis.

Treatment	Cost	Seizure-free	ICER*
Deterministic results			
BRV	JOD 3925	57.71%	
ESL	JOD 4139	28.70%	-JOD 737
LCM	JOD 3078	26.83%	JOD 2744
PER	JOD 5541	31.27%	-JOD 6113
Probabilistic sensitivity analysis			
BRV	JOD 3914	57.74%	
ESL	JOD 4136	28.64%	-JOD 761
LCM	JOD 3077	26.82%	JOD 2707
PER	JOD 5529	31.28%	-JOD 6100

BRV = brivaracetam, ESL = eslicabazepine, ICER = incremental cost-effectiveness ratio, LCM = lacosamide, PER = perampanel.

* Estimates are approximated to 2 decimal points.

### 3.2. Sensitivity analysis

#### 3.2.1. PSA ICER

As shown in Table 3, the confirmatory analysis showed consistency with the deterministic analysis. In which, the incremental gain in CR with BRV was higher by 29.1%, 30.9%, and 26.5% compared to ESL, LCM, and PER, respectively. Despite these higher gains in CR with BRV versus all other AEDs in this economic evaluation, BRV was associated with lower cost when compared to ESL and PER at saving ICER of -JOD 761 per 1% CR achieved and -JOD 6100 per 1% CR achieved, respectively. However, BRV was associated with the incremental ICER of JOD 2707 per 1% of CR achieved compared with LCM.

**Figure 2.**
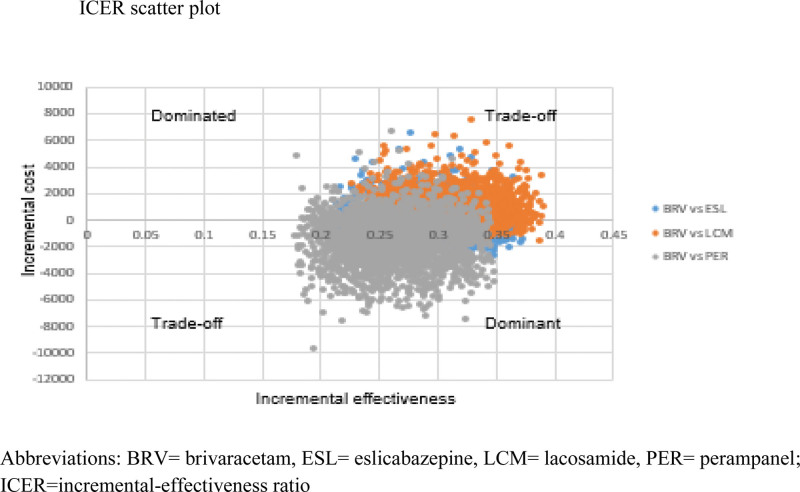
ICER scatter plot. ICER = Incremental Cost-Effectiveness Ratio.

Figure 2 shows the PSA iterations simulated by assuming ranges for the uncertain parameters like the transitional probabilities, cost of treatments, cost of healthcare services, and discount rates. In this figure, BRV was colored in 3 lines to present its location when a pairwise comparison was conducted between BRV and each AED. The blue-colored iterations represent the location of BRV versus the ESL (assuming ESL is at the reference point of this ICER plane); the orange-colored iterations represent the location of BRV versus the LCM (assuming LCM is at the reference point of this ICER plane); and the gray-colored iterations represent the location of BRV versus the PER (assuming PER is at the reference point of this ICER plane). The PSA iterations for BRV versus ESL were mainly in the cost-saving quadrant (lower right quadrant) by 63%. The iterations of BRV versus LCM were mainly in the trade-off quadrant (upper right quadrant:) by 76%. On the other hand, 19% of the BRV versus PER iterations were in the trade-off quadrant and 81% were in the cost-saving quadrant (lower right quadrant).

**Figure 3.**
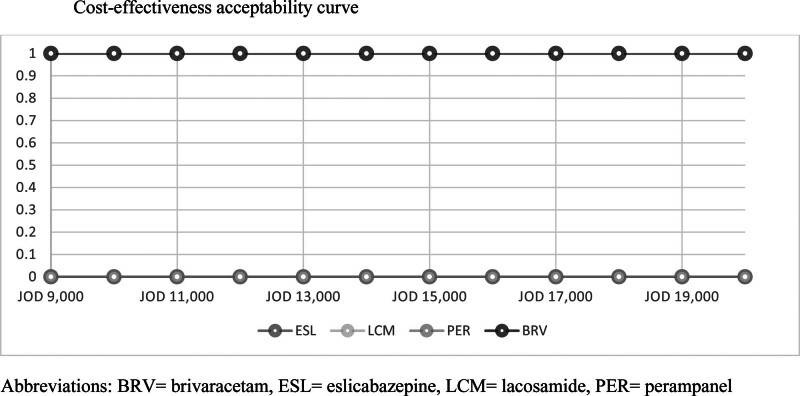
Cost-effectiveness acceptability curve.

Figure 3 shows the cost-effectiveness acceptability curve that started at a willingness-to-pay of JOD 9000 per 1% of CR. The BRV was cost-effective at this threshold compared to all other AEDs. In our economic evaluation, any WTP below the JOD 9000, BRV was cost-effective and associated with certainty of 100% compared to ESL and PER; and 80% compared to LCM.

## 4. Discussion

Epilepsy is a condition that is indicative of the presence of recurrent seizures that lack an immediate identifiable cause. Age, genetics, and origin are the most common variations in this disorder, which encompasses a wide range of risk factors and complex symptoms.^[16,17,19]^ This economic evaluation examined the cost-effectiveness of BRV compared to other third-generation AEDs for the treatment of pharmacoresistant FOS in Jordan. In our economic evaluation, BRV was a cost-saving option compared to ESL and PER; and cost-effective compared to LCM at the PSA ICER of JOD 2704 per 1% of CR achieved. This incremental cost for BRV compared to LCM is justified due to the reduced cost of LCM in Jordan. In contrast to the PER and ESL, which are available under a single brand name (FYCOMPA) and (ZEBINIX), LCM is available in Jordan in a variety of generic forms and doses.

The medication under investigation in this study (BRV) proved its effectiveness in treating pharmacoresistant FOS in clinical trials as an add-on treatment.^[20–22]^ For patients with pharmacoresistant epilepsy it was found that the use of combination of traditional AEDs does not benefit the patients, thus, adding newer AEDs such as BRV has become an acceptable clinical practice.^[4,5]^ There is limited research on the effectiveness of newer AEDs for the treatment of pharmacoresistant FOS with a limited number of head-to-head trials of several newer AEDs. Thus, the only objective comparison available is the indirect comparison obtained from network meta-analysis studies.^[4,9,10]^ Network meta-analysis enables the comparison of multiple treatments simultaneously in a single analysis by combining direct and indirect evidence within a network of randomized controlled trials.

Charokopou et al conducted a network meta-analysis that included 65 randomized controlled trials of patients with FOS.^[9]^ The findings of this network meta-analysis suggested that the efficacy, safety, and tolerability outcomes of the included AEDs, including BRV, are relatively similar.^[9]^ Lattanzi and colleagues conducted another network meta-analysis study with stricter inclusion criteria.^[10]^ They compared BRV, cenobamate (CNB), ESL, LCM, and PER as adjunctive treatment for FOS.^[10]^ In their study, Lattanzi and colleagues ranked CNB as the most effective, and found BRV and LCM to be the most well-tolerated among the other options.^[10]^ In 2024, the most recent network meta-analysis study examined the effectiveness, tolerability, and safety of third-generation AEDs in treating treatment-resistant focal-onset seizures.^[4]^ This network meta-analysis included 7 third-generation AEDs, namely BRV, ESL, LCM, PER, CNB, retigabine, and rufinamide.^[6]^ In this network meta-analysis study, BRV ranked first for both serious adverse effects and seizure freedom. Upon considering dosages, BRV at 100 mg/day emerged as the most effective option for seizure freedom.^[4]^ Additionally, BRV was the least likely medication to induce withdrawal, ranking second after placebo. Important to note that medications were compared at different doses and the results changed accordingly. It is worth mentioning that in Jordan, CNB, retigabine, and rufinamide are not available as a treatment option for patients with FOS. As a result, alternative AEDs are prescribed by healthcare providers in accordance with international and local guidelines to ensure that patients receive proper and effective management for their seizures.

Third-generation AEDs often share a common characteristic, such as being used in pharmacoresistant epilepsy cases as adjunctive treatment, partly due to increased cost. This increases the need for an economic evaluation of those medications. Comparing the results of cost-effectiveness and cost-utility studies for epilepsy treatments is challenging because of the variations in the economic evaluation models and parameters used in these studies.^[23]^ Wijnen and colleagues conducted a systematic review of economic studies of epilepsy treatments.^[23]^ In their study, Wijnen and colleagues included 29 articles (73%) on pharmacological interventions, including AEDs. Most of the examined third-generation AEDs studies did not calculate the ICER, and given that the study was published in 2016, many newer medications were not evaluated economically yet, such as CNB and BRV. Nonetheless, it is crucial to differentiate between studies that have conducted a cost-utility analysis (CUA) and those that have conducted a cost-effectiveness analysis. Within the context of the CUA, the outcomes are expressed as the cost per QALY gained. Conversely, the cost-effectiveness analysis presents the results in terms of the cost per clinical outcome measure. The availability of WTP for QALYs in many countries, such as the Netherlands (EUR €20,000 and EUR €80,000), makes it easier to assess if the intervention is cost-effective, unlike clinical outcomes.^[23]^ Wijnen and colleagues acknowledged that while the methodological quality of the included studies was generally considered acceptable, some studies showed significantly lower quality.^[23]^ They also noted a lack of comparability between epilepsy research due to the nature of treatments and the different types of epilepsy investigated.^[23]^ Besides, Wijnen and colleagues provided useful recommendations that underscores the need for a more consistent method for conducting economic assessments in the field of epilepsy such as creating a benchmark case that establishes a framework for future research to ensure consistency in conducting economic evaluations.^[23]^

Similar to the methodology implemented in our study, the pharmacoeconomic assessment conducted by Martinez et al used comparative results from meta-analyses to build on economic evidence.^[11]^ Martinez et al conducted CUA because of availability of patient preferences data in Spain.^[11]^ Network meta-analysis is very useful source of information to be employed in building a Markov model for assessing BRV’s cost-effectiveness, compared with other third-generation AEDs in Jordan, against pharmacoresistant focal-onset seizures, with limited clinical data and information on patient preference. Network meta-analysis provides integration of data from several sources and thus offers a more complete comparison of BRV against a range of AEDs by synthesizing indirect evidence where direct comparisons are not available. This methodological approach enhances the robustness and generalizability of the model and therefore enables more informed decision-making, despite the scarcity of local data. It would help the Markov model to better reflect the clinical and economic outcomes of BRV use in Jordan and would optimize the treatment strategies for pharmacoresistant FOS by including a wider spectrum of evidence.

Due to the absence of data on patient preference for the Jordanian FOS patients, we intended to conduct our economic evaluation in light of the percentage of patients who achieved CR. Moreover, third-generation AEDs used in our study are comparable to the comparators used in Martinez et al, except for ZNS, which is not available in the Jordanian market. Knowing the nature of the disease, it was expected to further complicate and cause side effects, hence, increasing the cost of the treatments. Therefore, we projected the cost of several healthcare services, such as emergency visits, inpatient care, visits to health providers, and the use of EEGs. The same applies to the drugs that are to be used, the third-generation AEDs, which again will have varying degrees of side effects. Similar to the study by Martinez et al, we included the most commonly reported side effects, namely ataxia, dizziness, fatigue, nausea, and somnolence.^[11]^ Furthermore, in our model, we have accounted for the cost of side effects management, such as the use of acetazolamide and cinnarizine for treating ataxia and dizziness, respectively. The results from Martinez et al suggested that BRV is the most favorable choice compared to the other options.^[11]^ However, in terms of cost utility, the disparities are less prominent because BRV has a higher occurrence of negative effects compared to the other treatments.^[11]^

The findings of this study are significant as they inform decision-making related to antidepressant therapies, facilitate the reevaluation of the value of both existing and emerging treatments within the Jordanian formulary, and support purchasers in assessing reimbursement policies and establishing priority settings.

In Finland, Väätäinen et al assessed the cost-effectiveness of BRV compared to PER for patients with FOS.^[24]^ The model was constructed on a 5-years’ time horizon with a 3% discount per year. Väätäinen et al study adopted the UK threshold (EUR €25,358 and EUR €38,036 per QALY) due to the absence of WTP thresholds in Finland. In comparison with PER, BRV exhibited a 71% and 80% probability of being cost-effective at a WTP of €25,358 per QALY gained.^[24]^ Despite the fact that Väätäinen et al and our study were conducted using different approaches, both studies have shown that BRV was cost-effective, and cost-saving compared to PER when used for treating FOS in simulated Jordanian cohort.

Based on our study findings, it recommended that decision-makers and healthcare professionals working in Jordan consider BRV a therapy to be placed in priority order in the treatment algorithms of pharmacoresistant FOS. Although BRV had a more prominent incremental cost than LCM, its better clinical efficacy makes it justifiable for administration with high importance to reaching maximal seizure control in some patients. These are further supported by strong PSA data that found BRV to maximize patient outcomes within currently available health care resources should a change in its place in national guidelines take place.

This study is not without limitations. This economical evaluation did not include real patient data, as the local patient-level data and patient-reported outcomes (utilities) specified for Jordanian epileptic patients are lacking. Furthermore, some important information regarding the disease cannot be captured, such as the costs of all possible side effects, medication adherence, productivity, and patient time loss. In addition, this economical evaluation did not include all third-generation AEDs; however, it did include all comparators to BRV registered in Jordan. Similarly, only the most common side effects were accounted for in the simulation model. That said, despite the localization made using the available data in Jordan, this economic evaluation showed a significant degree of consistency with previously literature.^[11]^ Despite the fact that the results of network meta-analysis are highly beneficial to healthcare providers and decision-makers. This does not exclude the necessity of conducting head-to-head trials and suggests that NMAs should not serve as the only basis for treatment recommendations, as they are incapable of accounting for unmeasured confounding factors and population heterogeneity.^[25]^ This study was based on phase 3 clinical trial data. Future pharmacoeconomic evaluations should incorporate real-world evidence to generate more robust ICER estimates, thereby enhancing decision-making regarding the comparative effectiveness of treatment alternatives and the associated economic implications.

## 5. Conclusion

Compared to ESL and PER, BRV was associated with cost-savings. Compared to LCM, the BRV was cost-effective at the World Health Organization recommended willingness-to-pay threshold of 3× of Jordanian gross domestic product per capita. Our findings, which suggest that BRV is the optimum third-generation AED for the treatment of pharmacoresistant FOS, should encourage healthcare payers to consider the most cost-effective and cost-saving option once it is available in Jordan.

## Author contributions

**Conceptualization:** Shiraz Halloush, Nimer S Alkhatib.

**Data curation:** Shiraz Halloush, Nimer S Alkhatib.

**Formal analysis:** Shiraz Halloush, Nimer S Alkhatib.

**Funding acquisition:** Nimer S Alkhatib.

**Investigation:** Shiraz Halloush, Nimer S Alkhatib, Osamah M. Alfayez, Omar S. Alkhezi, Rawan Al Froukh, Farah Radaideh, Abdallah Y Naser.

**Methodology:** Shiraz Halloush, Nimer S Alkhatib.

**Project administration:** Shiraz Halloush, Nimer S Alkhatib.

**Resources:** Shiraz Halloush, Nimer S Alkhatib, Osamah M. Alfayez, Omar S. Alkhezi, Rawan Al Froukh, Farah Radaideh, Abdallah Y Naser.

**Software:** Shiraz Halloush, Nimer S Alkhatib.

**Supervision:** Shiraz Halloush, Nimer S Alkhatib.

**Validation:** Shiraz Halloush, Nimer S Alkhatib, Abdallah Y Naser.

**Visualization:** Shiraz Halloush, Nimer S Alkhatib.

**Writing – original draft:** Shiraz Halloush, Nimer S Alkhatib.

**Writing – review & editing:** Shiraz Halloush, Nimer S Alkhatib, Osamah M. Alfayez, Omar S. Alkhezi, Rawan Al Froukh, Farah Radaideh, Abdallah Y Naser.

## Supplementary Material


