# Supplementary material for: Economic evaluation of brivaracetam in the adjunctive treatment of patients with focal-onset seizure in Jordan
Source: Medicine (Baltimore). 2025 Oct 31;104(44):e45347. doi: 10.1097/MD.0000000000045347 (PMC12582735; doi:10.1097/MD.0000000000045347)
Supplement: Supplementary file 1 [file medi-104-e45347-s001.docx]

Table S1. Dosing of ASMs

| **ASM** | **Dosing according to the SmPC** | **Median first cycle** | **Median subsequent cycles** |
| --- | --- | --- | --- |
| BRV | 50 mg PO/IV q12hr initially; based on individual patient tolerability and therapeutic response, adjust dose between to 25-100 mg PO/IV BID (50-200 mg/day) | 100 mg/day | 125 mg/day |
| ESL | Initial: 400 mg PO qDay/Adjunctive therapy: 1600 mg/day should be considered in patients who did not achieve a satisfactory response with 1200 mg/day | 800 mg/day | 1200mg/day |
| LCM | Initial: 50 mg PO/IV q12hr; based on response and tolerability, increase dose at weekly intervals by 50 mg PO/IV BID; up to a recommended dose of 100-200 mg BID (200-400 mg/day | 100mg/day | 300mg/day |
| PER | In absence of concomitant enzyme-inducing antiepileptic drugs (AEDs): 2 mg PO qHS initially; increase by 2 mg/day increments in at least weekly intervals based on clinical response and tolerability to 4-8 mg qHS; maintenance 8-12 mg/day | 2mg/day | 10mg/day |

Abbreviations: BRV= brivaracetam, ESL= eslicabazepine, LCM= lacosamide, PER= perampanel; ASM= antiseizure medication
